# The Impact of Maternal Obesity on the Duration of Labor Stages in Dinoprostone-Induced Vaginal Delivery

**DOI:** 10.3390/jcm14093209

**Published:** 2025-05-06

**Authors:** Neslihan Bezirganoglu Altuntas, Yesim Bayoglu Tekin

**Affiliations:** Department of Obstetrics and Gynecology, Trabzon Kanuni Training and Research Hospital, Maras Street, Ortahisar 61000, Türkiye

**Keywords:** dinoprostone, labor induction, body mass index, vaginal delivery, labor duration

## Abstract

**Objective:** Dinoprostone vaginal inserts are widely used as one of the primary pharmacological methods for labor induction. In this study, we aimed to investigate whether BMI affects the duration of the different phases of labor in pregnant women undergoing vaginal delivery induced with slow-release dinoprostone. **Methods:** A prospective study was conducted on pregnant women at a tertiary maternity hospital between August 2021 and February 2023. Patients were categorized into three groups according to BMI: normal-weight, overweight, and obese. The duration of total labor and each phase of induced labor was recorded. Multivariate analysis was used to determine the association between maternal obesity and the duration of each phase of the labor process. **Results:** The final analysis included 205 women who received slow-release dinoprostone for labor induction. The mean maternal age was significantly lower in the normal-weight group (*p* < 0.01). The obese group showed a higher need for oxytocin augmentation and had a higher median infant birth weight compared to the normal and overweight groups. After adjusting for confounders, multivariate linear regression analysis showed that the duration of the latent phase of labor did not differ between the groups. However, the duration of the active phase of labor and total induced labor were significantly longer in the obese group. **Conclusions:** An increased BMI in pregnant women is associated with a longer active phase and overall labor duration during dinoprostone-induced delivery.

## 1. Introduction

Since the historical milestone of discovering the uterus’s response to oxytocin and prostaglandin F2a over the century, pharmacological labor induction has become a common intervention in modern obstetrical practice [1]. A Cochrane systemic review in 2018, which included 20 randomized controlled studies with 9960 infants, confirmed that induction of labor (IOL) significantly reduced perinatal deaths compared to expectant management in term and post-term pregnancies (95% confidence interval [CI] 0.14–0.78) [2].

The Bishop score is the major factor in estimating successful labor induction. The duration from induction to delivery is crucial; extended labor periods are linked to increased rates of infection, maternal distress, supplementary oxytocin usage, and especially high rates of emergency cesarean sections [3]. Prostaglandin analogues play a key role in cervical ripening and parturition, including enhancing uterine contractility by sensitizing the myometrium to the effects of endogenous or exogenous oxytocin [4,5]. Therefore, dinoprostone (PGE_2_) and misoprostol (PGE_1_) are widely used worldwide for the initiation and/or continuation of cervical ripening in term pregnancies undergoing labor induction [6].

Studies have shown that obese women are more likely to undergo IOL compared to normal-weight women [7]. It has been suggested that the higher rate of post-term pregnancies and the associated co-morbidities such as preeclampsia and gestational diabetes contribute to the greater need for IOL in obese women [8]. Carhall et al. showed that obese women undergoing IOL spend a longer time from admission until the start of active labor than normal-weight women [9]. However, findings regarding the association between duration of active labor, >5 cm cervical dilation to delivery, and maternal body mass index (BMI) have been inconsistent across several studies [8,10,11,12]. Moreover, most studies used misoprostol for IOL, which showed a different efficacy than dinoprostone [13]. Since the increased duration from induction to vaginal delivery might be associated with increased morbidities in both mother and infant, assessing the risk of prolonged labor in obese pregnant women is crucial for determining appropriate interventions.

Thus, in this study, we aimed to investigate whether maternal BMI is associated with the duration of the total, latent, and active phases of labor induced with dinoprostone vaginal inserts in women undergoing vaginal delivery.

## 2. Materials and Methods

This prospective, observational cohort study was conducted between August 2021 and February 2023 at Kanuni Education and Research Hospital, Trabzon, Türkiye. Written informed consent was obtained from the patients. The participants were informed of their right to withdraw from the study at any point without consequences. The study was conducted in accordance with the Declaration of Helsinki and approved by the ethics committee of the hospital (26 November 2020-2020/47). Pregnant women were enrolled consecutively from all eligible women who presented for labor induction with dinoprostone at our hospital during the study period. Participants were included in the study based on the following criteria: (i) maternal age between 18 and 40 years, (ii) singleton pregnancy, (iii) at least 37 weeks 0 days of gestation by the first day of the last menstrual period, (iv) unfavorable cervical status defined as Bishop score (BS) of <5, (v) intact membranes, (vi) reactive non-stress test (NST), and (vii) cephalic presentation. Participants were excluded if they had multiple pregnancies, a history of cesarean section, abnormal NST results, breech, transverse, or oblique fetal presentation, contraindications to labor or vaginal delivery, additional maternal diseases, suspected fetal anomalies, or a known sensitivity to dinoprostone.

All patients undergoing IOL received dinoprostone in the form of a slowly released vaginal insert (Propess^®^), containing 10 mg of dinoprostone. The insertion procedure followed the manufacturer’s instructions, with the device placed high into the posterior vaginal fornix by an obstetrician, either with or without a speculum. The fetal heart rate (FHR) was monitored 30 min before and 1 h after insertion. Continuous cardiotocography (CTG) and vaginal examinations were performed every 2–3 h. The vaginal insert remained in place for a maximum of 12 h or when the active phase of labor began. If the insert was expelled within the first 12 h and the patient had no contractions or still had an unfavorable cervix, another insert was inserted and left in place for an additional maximum of 12 h. Intravenous oxytocin was administered as needed, at the discretion of the obstetrician, to continue the induction if regular uterine contractions were deemed insufficient following the removal of dinoprostone. Oxytocin was administered according to a standard regimen regardless of BMI. The infusion started at 2 mU/min and was increased by 2 mU/min every 20 min, up to a maximum of 20 mU/min, depending on uterine contractility.

Data on maternal characteristics, including age, parity, gestational week, weight, height, body mass index (BMI), date of last menstrual period, baseline Bishop score, oxytocin augmentation necessity and timing, infant birth weight (kg), and specific indications (such as oligohydramnios, intrauterine growth restriction (IUGR), and postmaturity) were recorded by the study team. Gestational age was categorized as <39 weeks, 39–41 weeks, and >41 weeks. Maternal BMI was calculated by trained nurses based on maternal height and weight measurements taken during admission to labor. Patients were categorized into three groups according to BMI, classified per the World Health Organization definitions: group 1 (normal-weight): 18.6–24.9 kg/m^2^, group 2 (overweight): 25–29.9 kg/m^2^, and group 3 (obese): ≥30 kg/m^2^. Underweight women were excluded from the study. For multivariate analysis, we further subdivided the patients into two groups: the non-obese group (BMI ≤ 29.9 kg/m^2^) and the obese group (BMI ≥ 30 kg/m^2^). Parity was distinguished as primiparous or multiparous. Total labor duration was defined as the interval from the start of induction to the time of birth. Latent labor (LL) was defined as the interval from the start of induction to cervical dilation > 5 cm. Active labor (AL) was defined as the period from cervical dilation > 5 cm to the time of birth [14]. Our objective was to compare the duration of total labor and each phase of induced labor using dinoprostone vaginal inserts between obese and non-obese patients.

### Statistical Analysis

The data were analyzed using SPSS version 25.0 (SPSS Inc., Chicago, IL, USA). Continuous variables were presented as mean ± standard deviation (SD) for normally distributed data or as median with interquartile range (IQR) for non-normally distributed data. Categorical variables were summarized as counts and percentages. For comparisons between the three BMI groups, one-way ANOVA was used for normally distributed continuous variables, and the Kruskal–Wallis test for non-normally distributed variables. Categorical variables were compared using Pearson’s chi-square test or Fisher’s exact test, as appropriate. Multivariate linear regression was used to determine the association between BMI and each phase of induced labor, separately comparing obese and non-obese groups. The analysis was adjusted for confounding variables, including maternal age, gestational age, parity, Bishop score, and birth weight. Entry into the multivariate model was conditional on a *p*-value of 0.2 in univariate analysis. For all statistical analyses, a *p*-value < 0.05 was considered significant. A previous study reported a mean induction-to-delivery time of 17.72 ± 7.3 h in non-obese women and a weighted mean of 20.97 ± 9.53 h in obese women [11]. Based on these findings, we calculated the required sample size using an effect size of 0.3, an alpha error of 0.05, and a statistical power of 0.80. The minimum required sample size was 96 patients for the obese and non-obese groups.

## 3. Results

**Figure 1:**
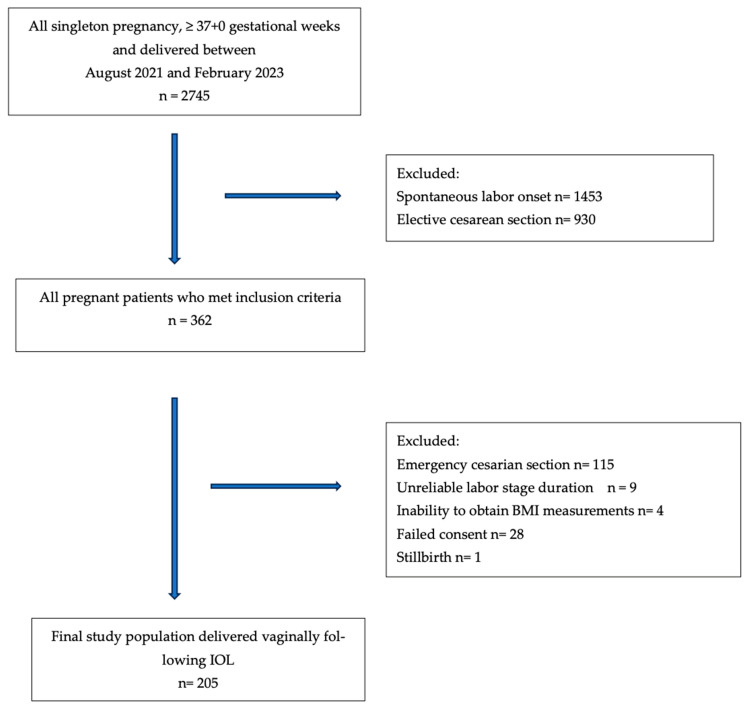
Flow chart of the study population.

During the study period, a total of 362 patients who met the inclusion criteria and underwent labor induction with a dinoprostone slow-release vaginal insert were included. Of these, 247 patients delivered vaginally following IOL. The emergency cesarean section rate was 24.3% (*n* = 9) for normal-weight patients, 33.9% (*n* = 40) for overweight patients, and 39.9% (*n* = 67) for obese patients. Forty-two patients were excluded from the study due to uncertain documentation of labor stage duration, inability to obtain BMI measurements, or failed consent. The final analysis included 205 pregnant women who were classified based on their BMI at the time of labor admission. Among them, 28 (13.6%) were classified as normal-weight, 76 (37.1%) as overweight, and 101 (49.3%) as obese (Figure 1).

The characteristics of the study population are summarized in Table 1. The mean maternal age was significantly lower in the normal-weight group compared to the other groups (*p* < 0.01). There were no significant differences in parity between the groups (*p* = 0.38). While the incidence of delivery at >41 gestational weeks increased with higher maternal BMI (21.4%, 31.6%, and 40.6%, respectively), the median gestational age at delivery did not statistically differ between the groups (*p* = 0.31). The obese group showed a higher need for oxytocin augmentation and had a higher median infant birth weight compared to the normal and overweight groups (*p* = 0.022 and 0.029, respectively).

A comparison of the duration of each phase of induced labor between the groups is presented in Table 2. The time between the start of induction and the active phase of labor was borderline, significantly higher in the obese group (*p* = 0.048). Furthermore, the obese group had prolonged active labor and total labor duration compared to the other groups (*p* = 0.02 and 0.009, respectively).

After adjusting for maternal age, gestational age, parity, Bishop score, birth weight, and oxytocin use, multivariate regression analysis showed that the duration of the latent labor did not differ between the obese and non-obese groups (*p* = 0.856). However, obesity was significantly associated with longer active labor duration (B = 3.14, 95% CI: 1.205–6.332, *p* < 0.01) and total induced labor duration (B = 3.54, 95% CI: 1.457–7.057, *p* = 0.01) (Table 3).

## 4. Discussion

The interval between the induction of labor and subsequent vaginal delivery is crucial for reducing the risk of cesarean delivery and adverse maternal and neonatal outcomes. The efficacy of dinoprostone vaginal inserts in labor induction has been well documented through various studies involving term pregnancies [6,15]. Nonetheless, this prospective cohort study is among the few that evaluate the duration of each phase of the labor process in relation to maternal BMI in women undergoing labor induction with dinoprostone vaginal inserts. Our findings indicate that the duration of the latent phase of induced labor did not differ between the obese and non-obese groups. However, the duration of the active phase of labor and the total induced labor duration were significantly longer in the obese group. Furthermore, the rate of oxytocin requirement increased significantly with a higher maternal BMI in induced labor with dinoprostone.

Increased maternal weight was associated with a longer latent phase of labor in our study. However, the duration of the latent phase of labor did not significantly differ by BMI category after adjusting for confounders. This finding is consistent with that of Hiersberg et al., who did not observe a difference in latent labor duration by BMI category in women with vaginal deliveries who underwent IOL [16]. In contrast, another study reported that labor progression in overweight and obese women was significantly slower than in normal-weight women before reaching 6 cm of cervical dilation [17]. They speculated that additional soft-tissue deposits in the maternal pelvis might narrow the birth canal diameter, necessitating more time and stronger contractions to progress. Furthermore, a large Swedish study involving 15,259 pregnant patients categorized by pre-pregnancy body weight indicated that maternal BMI had a greater impact on the time from admission until the start of active labor, with the duration increasing progressively with higher maternal BMI in induced labor [9]. Our study was restricted to patients who had vaginal deliveries induced by dinoprostone, used a different cervical dilation length for the latent phase of labor, and classified patients by body weight at the time of induction. The differences in methodology may explain the variations in the findings.

The impact of obesity on the duration of the active phase of labor and the overall labor duration has been previously shown [11,18,19]. In line with our findings, a previous study demonstrated that maternal obesity is associated with active phase labor dysfunction, particularly the arrest of dilatation [18]. Similarly, another study found that the total duration of induced labor increases with a higher maternal weight at delivery [11]. Furthermore, a large Misoprostol Vaginal Insert Trial assessing the outcomes of over 1200 women based on BMI category revealed that obese women experienced a longer time interval until delivery, required more oxytocin, and had a higher cesarean delivery rate in induced labor [19]. One plausible explanation for these findings is provided by Zhang et al., who suggested that obesity might impair uterine contractility during labor [20]. Their study showed that the myometrium of obese women contracted with less force and frequency and exhibited reduced [Ca^2+^] flux compared to that of normal-weight women [20]. Consistent with our findings, a dose-dependent relationship between increasing maternal BMI and higher oxytocin requirements has also been indicated in previous studies [21,22,23]. Garabedian et al. suggested that decreased receptor sensitivity to oxytocin in individuals with a higher BMI could be another contributing factor [22]. Another reason could be the increased volume of distribution observed in obese women, which might diminish the efficacy of oxytocin and lead to a reduced tissue response during labor induction [23]. This pharmacokinetic alteration may not be limited to oxytocin. In obese patients, the increased volume of distribution may also affect dinoprostone, potentially leading to reduced local drug concentrations and diminished clinical effects. We used a standard dose regimen of slow-release dinoprostone vaginal inserts for all patients. Whether dinoprostone is subject to an increased volume of distribution in obese patients requires further investigation.

Our study has several limitations. Firstly, it was conducted in a single center with a relatively small sample size. The higher proportion of obese women in our study may be attributed to the fact that our institution is a tertiary referral center that receives a greater number of high-risk pregnancies. Secondly, we recorded the BMI at admission to labor, and therefore did not account for the effect of pregestational BMI and weight gain during pregnancy on labor duration. Furthermore, the frequency of cervical examinations was standardized across all patients to ensure consistency in recording labor progression. However, the exact onset of active labor may still vary depending on the timing of these assessments. The study did not use interval-censored regression analysis to statistically adjust for these time-dependent variations.

In conclusion, our study revealed that maternal obesity, defined as a BMI of ≥30, is associated with a prolonged active phase and total labor duration, as well as a higher oxytocin requirement in induced labor. We suggest that more personalized care may be required for pregnant women with higher BMIs to manage their labor more effectively.

## Figures and Tables

**Table 1:** General characteristics of the study groups.

	Normal Weight (*n* = 28)	Overweight (*n* = 76)	Obese (*n* = 101)	*p*-Value ^a^
Maternal age (years) (SD)	23.5 (20–26)	28 (24–31)	27 (25–32)	<0.01
Parity, *n* (%)				
Nulliparous	20 (71.4)	54 (71.1)	62 (61.4)	0.38
Parous	8 (28.6)	22 (28.9)	39 (37.6)	
Gestational age (week), median (IQR)	39 (38–40)	40 (38–41)	40 (38–41)	0.42
Gestational age (*n*, %)				
<39 weeks	8 (28.6)	20 (26.3)	28 (27.7)	
39–41 weeks	14 (50)	32 (42.1)	32 (31.7)	0.31
41 weeks	6 (21.4)	24 (31.6)	41 (40.6)	
BMI, kg/m^2^, median (IQR)	23.1 (21.6–24.2)	27.9 (26.7–29.2)	32.55 (31.2–35.2)	<0.01
Oxytocin augmentation (*n*,%)	8 (28.6)	30 (38.5)	58 (58.6)	0.022
Oxytocin time, h, median (IQR)	7 (2–9)	6 (3–7)	6 (3.75–7.35)	0.017
Baseline Bishop score, median (IQR)	3 (1–4)	3 (1–4)	3 (1–4)	0.178
Infant weight, gram, median (IQR)	2955 (2800–3140)	3220 (3000–3420)	3325 (3030–3600)	<0.01
Birth weight (*n*,%)				
<2500 g	6 (21.4)	4 (5.3)	8 (7.9)	0.029
2500–4000 g	20 (71.4)	70 (9.2)	82 (81.2)	
>4000 g	2 (7.1)	2 (2.6)	11 (10.9)	
Indications for induction, *n* (%)				
Oligohydramnios	6 (21.4)	26 (34.2)	24 (23.8)	
IUGR	6 (21.4)	2 (2.6)	8 (7.9)	0.059
Postmaturity	10 (35.7)	26 (34.2)	44 (43.6)	
Fetal reasons	3 (10.7)	12 (15.8)	12 (11.9)	
Other	3 (10.7)	10 (13.2)	13 (12.9)	

Plus–minus values are mean ± standard deviation; SD, standard deviation; IQR, interquartile range; BMI, body mass index; IUGR, intrauterine growth restriction; ^a^ *p*-values < 0.05 were considered significant.

**Table 2:** Comparison of each phase of induced labor between the groups.

Phases ^a^	Normal Weight (*n* = 28)	Overweight (*n* = 76)	Obese (*n* = 101)	*p*-Value ^b^
Latent labor time, hours	7 (4–9)	8 (5.5–10)	8.50 (6.5–12)	0.048
Active labor time, hours	4.5 (3–7)	6 (4–13)	9 (4–16.5)	0.02
Total labor time, hours	11.25 (8–14.38)	14 (8.5–20.5)	17 (10.63–22.75)	0.009

^a^ Median (interquartile range); ^b^ *p*-values < 0.05 were considered as significant.

**Table 3:** Multivariate linear regression analysis to estimate the effect of obesity for each phase of induced labor.

	*B* Coefficient ^a^	95% CI	*p* Value ^b^
Duration of latent labor (LL)	0.36	(0.012–0.855)	0.856
Duration of active labor (AL)	3.14	(1.205–6.332)	<0.01
Duration of total induced labor ^c^	3.54	(1.457–7.057)	0.01

^a^ *B* coefficient after adjustment for maternal age, gestational age, parity, Bishop score, birth weight, oxytocin use and treatment group; ^b^ *p*-values < 0.05 were considered significant; ^c^ Obese vs. non-obese group. CI, confidence interval.

## Data Availability

Data are contained within the article.
